# Correlation between amyloid deposits affecting renal compartments and glomerular filtration rate during renal biopsy in a renal amyloidosis case series

**DOI:** 10.1590/1414-431X20208625

**Published:** 2020-05-18

**Authors:** E.O. Fonseca, M.L.R. Caldas, P.J. Soares, J.R. Almeida

**Affiliations:** 1 Hospital Universitário Antônio Pedro, Universidade Federal Fluminense, Niterói, RJ, Brasil; 2 Programa de Pós-Graduação em Ciências Médicas, Universidade Federal Fluminense, Niterói, RJ, Brasil; 3 Departamento de Patologia, Universidade Federal Fluminense, Niterói, RJ, Brasil; 4 Departamento de Medicina Clínica, Disciplina de Nefrologia, Universidade Federal Fluminense, Niterói, RJ, Brasil

**Keywords:** Amyloidosis, Glomerular filtration rate, Amyloid deposits

## Abstract

Amyloidosis comprises a group of disorders that accumulate modified autologous proteins in organs, mainly the kidneys. Few studies have addressed the amyloid compartmental distribution and associated clinical outcomes. The aim of this study was to present a case series of renal amyloidosis correlating histopathological data with glomerular filtration rate (GFR) during kidney biopsy. We studied 53 cases reviewed by nephropathologists from 2000 to 2018 in a single kidney biopsy center in Brazil. GFR was estimated using the CKD-EPI formula. Cases were divided into Group A ≥60 and Group B <60 mL·min^−1^·(1.73 m^2^)^−1^ using the estimated GFR during kidney biopsy. Semiquantitative histopathological study was performed, including extension and distribution of amyloid deposits by compartments (glomeruli, tubulointerstitial tissue, and vessels). Statistical analyses were made to understand associations with lower GFR. No difference was seen for age, gender, proteinuria, hematuria, subtype of amyloid protein, arteriosclerosis, interstitial fibrosis/infiltrate, or glomerular and interstitial amyloid deposits. After a previous P value <0.1 in the descriptive analysis, the following variables were selected: globally sclerotic glomeruli, high blood pressure, and the extension of vascular amyloid deposition. A binary logistic regression model with GFR as the dependent variable showed history of hypertension and vascular amyloid to be robust and independent predictors of Group B <60 mL·min^−1^·(1.73 m^2^)^−1^. Beyond the histopathologic diagnosis of amyloidosis, a semiquantitative approach on renal biopsy could provide new insights. Vascular amyloid is an independent predictor of renal dysfunction in cases of renal amyloidosis.

## Introduction

Amyloidosis comprises a group of disorders that share the ability to accumulate modified autologous proteins in certain organs (1). Such proteins, or amyloid deposits, undergo changes in their structural conformation and become insoluble and alter the architecture and function of the compromised organ (2). As a rare disease, the epidemiology of amyloidosis in the general population is not well known (3), but the incidence of renal amyloidosis ranges from 0.7 to 4.8% in kidney biopsy studies worldwide (3–6), with this number approaching 2.3% in Brazil (7,8). Kidneys are often affected in systemic amyloidosis and the deposits can be detected in the glomeruli, tubulointerstitial tissue, or even blood vessels (9). Primary amyloidosis is related to blood cell dyscrasia and deposition of immunoglobulin light chains (AL), whereas secondary amyloidosis is associated with chronic inflammatory disorders and consequent deposition of protein A (AA) (10). Other less common types of amyloidosis include hereditary amyloidosis; however, it is possible to group such disorders as AL and non-AL using immunofluorescence for Kappa and Lambda light chains (4 [Bibr B05]). Immunofluorescence can be used to easily identify AL cases in fresh tissue provided from routine kidney biopsies, while immunohistochemistry for amyloid A protein requires a specific marker rarely available in pathology services.

The clinical presentation of renal amyloidosis is mainly characterized by proteinuria in patients older than 50 years of age. Many of these patients have histories of hypertension at diagnosis, likely as a comorbidity of nephrogenic origin. Loss of renal function may also be present and hematuria is occasionally reported (11). Despite the presence of amyloid deposits in the three renal compartments (glomeruli, tubulointerstitial tissue, and vessels), few authors have studied their compartmental histopathological distribution and possible associated clinical outcomes (9,12–16). Semiquantitative analysis is useful for measuring microscopy findings, but it has not been routinely applied for quantification of amyloid deposits in renal compartments (17). The objective of this study was to present a case series of renal amyloidosis correlating the histopathological data of the amyloid deposits with different glomerular filtration rates during renal biopsy.

## Material and Methods

We retrospectively studied patients with proven renal amyloidosis biopsies from 2000 to 2018 at the Antonio Pedro University Hospital (Federal Fluminense University/Niteroi), a public regional reference center to kidney biopsies (metropolitan region II) in Rio de Janeiro State, Brazil. The study was approved in accordance with Brazilian ethical standards and legislation on human studies (CAAE05460713.0.0000.5243). Paraffin blocks from renal amyloidosis cases were obtained from the pathology department archives to be reviewed depending on their availability or technical suitability. Serial sections, 3-μm thick, were newly stained with hematoxylin-eosin (HE), periodic acid Schiff (PAS), Masson trichrome (MT), and silver methenamine stain (PASM). Additional 7-μm-thick cuts were stained with Congo red using the Highman technique and light microscopy analysis was performed under dark field and polarized light. Information about the previously performed immunofluorescence microscopy on frozen tissue was obtained from the renal biopsy reports when available to classify the cases as AL or non-AL, as previously described in literature (4). Immunofluorescence showed AL amyloid positivity for the corresponding light chain (Kappa or Lambda) in a smudgy pattern. Conversely, in non-AL, immunofluorescence showed no specific staining. Clinical and laboratory data were also obtained from the medical records at the time of renal biopsy, including sex, ethnicity, age, 24-h proteinuria, presence or absence of hematuria, presence or absence of history of hypertension, and serum creatinine. Information on history of hypertension was obtained from the biopsy requirement sheet form sent to the Pathology Service. There was no detail on how long the patients were hypertensive but rather that they had hypertension with ongoing treatment prior to renal biopsy for diagnosis of amyloidosis.

**Figure 1:**
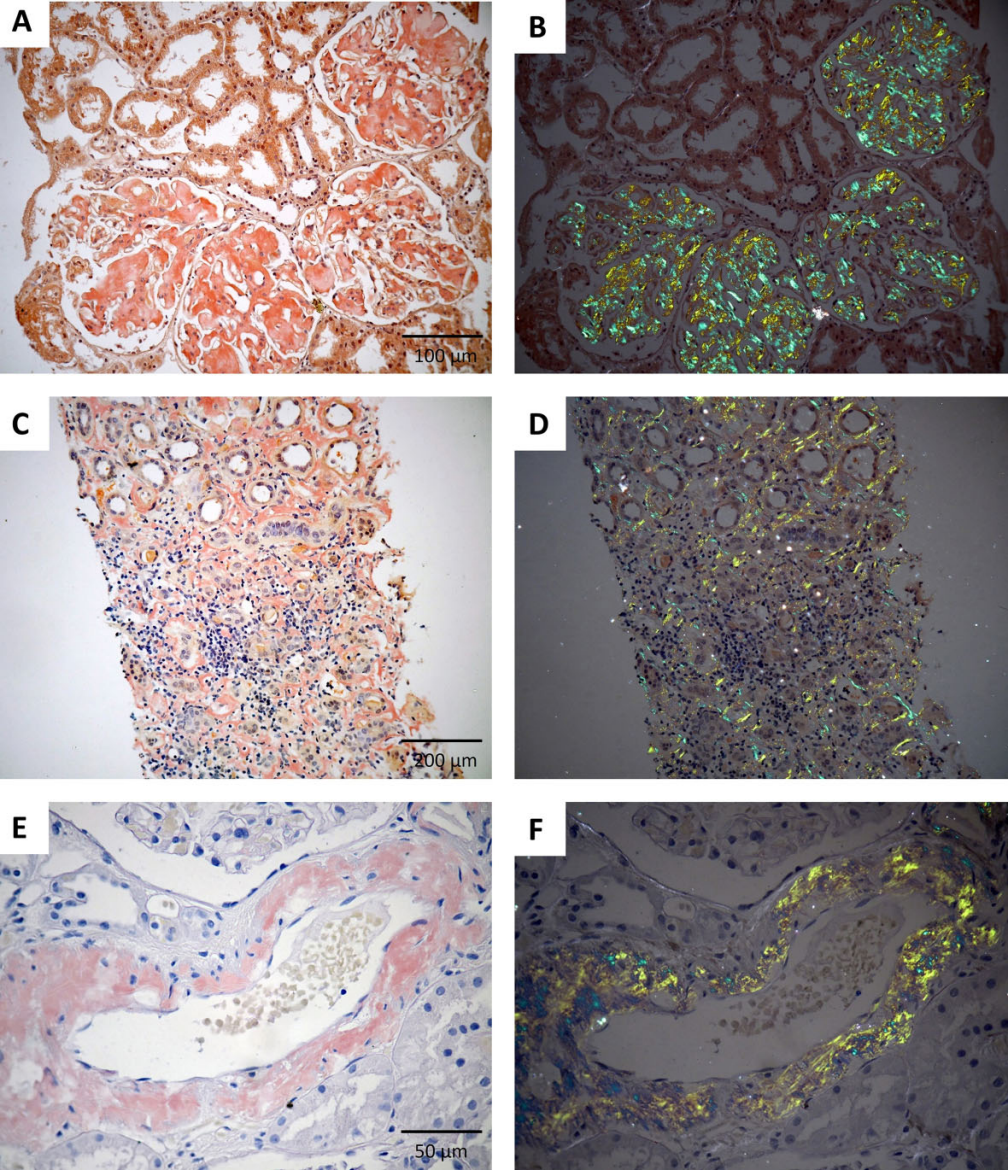
Representative photomicrographs illustrate amyloid deposits in renal compartments. **A** and **B**, glomeruli; **C** and **D**, tubulointerstitial tissue; **E** and **F**, vessels. On the left, amyloid deposits can be identified with Congo red. On the right, the same respective areas observed under dark field and polarized light, showing amyloid deposits with characteristic birefringent effect. Scale bars: **A** and **B**, 100 μm; **C** and **D**, 200 μm; **E** and **F**, 50 μm.

We semi-quantitatively categorized the degree of glomerular, tubulointerstitial, and vascular deposits and the degree of arteriosclerosis, interstitial fibrosis, and inflammatory infiltrate. For semiquantitative analysis, the extent of amyloid deposition in each of these compartments (glomeruli, tubulointerstitial tissue, and blood vessels) was classified as scores: 0) absent; 1) mild (less than 25%); 2) moderate (from 25 to 50%); and 3) severe (greater than 50%) (13,17). For practical purposes and categorical analysis, a score ≥2 (moderate to severe changes) was empirically chosen as a cutoff point representing a very consistent way of grading and estimating lesions. Of the total number of glomeruli present in each biopsy, we determined the percent of globally sclerotic glomeruli. These histopathological characteristics were collaboratively evaluated by two renal pathologists (EOF and MLRC) who considered the sample adequacy (total number of glomeruli), dye affinity of amyloid deposits (HE, PAS, MT, PASM), and birefringence (CR stained sections under polarized light). This approach allowed us to semi-quantitatively relate the scores to the presence of amyloid deposit within glomeruli, tubulointerstitial tissue, and blood vessels. Figure 1 illustrates the appearance of amyloid deposits in the renal tissue.

Renal function is reported by glomerular filtration rate (GFR), and estimated according to age, gender, and serum creatinine, according to the CKD-EPI equations for GFR (18,19). We divided the cases into two groups to separate cases with mild chronic renal insufficiency from those with moderate or severe chronic renal insufficiency: Group A: GFR above or equal to 60 mL·min^-1^·(1.73 m^2^)^-1^ (CKD stages I and II; mild CKD cases); and Group B: GFR below 60 mL·min^-1^·(1.73 m^2^)^-1^ (CKD stages III, IV, and V; moderate and severe CKD cases). As mentioned previously, this empirical approach was made because that cutoff would accurately represent an evident loss in renal function in group B, helping us to perform further statistical analysis and between-groups comparisons.

Data are reported as means±SD for continuous variables and percentages for categorical variables. We performed the Fisher’s exact test on categorical variables. For continuous variables whose normality could not be assumed, the non-parametric Mann-Whitney *t*-test was chosen. After selection of a previous P value <0.1 in the descriptive analysis as the first step, we performed a stepwise binary logistic regression model with GFR as dependent variable (outcome) to estimate odds ratio. A P value ≤0.05 was considered statically significant.

## Results

We investigated 53 histological cases of renal amyloidosis confirmed by two nephropathologists, from January 2000 to December 2018. We could not obtain an estimated GFR in four cases. Therefore, 49 cases were used in this study, comprising 53.1% males and 46.4% Caucasian, with a mean of age of 55.8±12.9 years. After division into two groups according to the e-GFR cutoff of 60 mL·min^-1^·(1.73 m^2^)^-1^ as explained previously, we obtained 17 patients in Group A and 32 patients in Group B. In Groups A and B, the e-GFR means±SD were 82.3±14.8 mL·min^-1^·(1.73 m^2^)^-1^ and 28.1±17.3 mL·min^-1^·(1.73 m^2^)^-1^, respectively.

There was no difference in age, gender, proteinuria, presence of hematuria, or presence of AL amyloid protein between Groups A and B. However, the percentages of globally sclerotic glomeruli and history of high blood pressure were significantly higher in Group B (P=0.01 and P=0.02, respectively). Glomerular sclerosis here refers to the collagen deposit, while glomerular amyloid deposits are characterized by the apple-green birefringence using Congo red staining. Histopathological semiquantitative scores were determined using a categorical cutoff score ≥2 as described in Methods. We found no statistical differences between Group A and Group B for arteriosclerosis, interstitial fibrosis, and interstitial inflammatory infiltrate.

Using Congo red staining associated to dark fields and polarized light, we applied the same scoring approach to quantify the extension of amyloid deposits in the renal tissue compartments. Interestingly, we found no differences in the extension of glomerular amyloid deposits and interstitial amyloid deposits. However, we found greater vascular amyloid deposition in a significantly higher percentage of cases in Group B (P=0.01). Table 1 shows the clinical and histopathological findings and comparisons between the two groups.

**Table 1:** Clinical, laboratory, and histological characteristics of renal amyloidosis patients according to chronic kidney disease stage groups at biopsy.

Parameters	Group A (≥60)	Group B (<60)	P value
Age (years), mean±SD (n)	53.2±15.4 (17)	57.2±11.3 (31)	0.35
Proteinuria (mg/24h), mean±SD (n)	6859±4888 (14)	5411±4528 (28)	0.36
Globally sclerotic glomeruli, % mean±SD (n)	3.9±6.8 (15)	12.9±16.8 (29)	0.01
Gender, male/female (% males)	10/7 (58.8%)	16/16 (50.0%)	0.76
Ethnicity, Caucasian/not (% Caucasians)	5/5 (50%)	8/10 (44.4%)	1.00
Hematuria, yes/no (% yes)	3/10 (23.1%)	12/16 (42.8%)	0.30
AL amyloid, yes/no (% yes)	9/6 (60.0%)	18/8 (69.2%)	0.73
History of hypertension, yes/no (% yes)	5/9 (35.7%)	20/7 (74.1%)	0.02
Arteriosclerosis score ≥2, yes/no (% yes)	0/15 (0.0%)	3/27 (10.0%)	0.54
Interstitial inflammation score ≥2, yes/no (% yes)	2/14 (12.5%)	8/21 (27.6%)	0.29
Interstitial fibrosis score ≥2, yes/no (% yes)	3/13 (18.8%)	6/23 (20.7%)	1.00
Glomerular deposits score ≥2, yes/no (% yes)	11/5 (68.7%)	19/10 (65.5%)	1.00
Vascular deposits score ≥2, yes/no (% yes)	2/14 (12.5%)	16/13 (55.2%)	0.01
Interstitial deposits score ≥2, yes/no (% yes)	1/15 (6.3%)	3/27 (10.0%)	1.00

Group A (≥60): GFR above or equal to 60 mL·min^-1^·(1.73 m^2^)^-1^ (CKD stages I and II). Group B (<60): GFR below 60 mL·min^-1^·(1.73 m^2^)^-1^ (CKD stages III, IV, and V). For continuous variables, the Mann-Whitney test was used and for categorical variables, Fisher’s exact test was used. CKD: chronic kidney disease; n: number; SD: standard deviation; AL: light chain amyloidosis. For categorical variables, we report the exact number of events (yes/no) by parameter in each line.

After a previous P<0.1 in the descriptive analysis, the following variables were selected: globally sclerotic glomeruli, high blood pressure, and extension of vascular amyloid deposition (Table 1). In the last step, a model of binary logistic regression model with GFR as the dependent variable revealed history of hypertension (OR=10.0; 95%CI=1.7-59.4; P=0.011) and vascular amyloid deposits (OR=11.9; 95%CI=1.6−89.7; P=0.016) to be robust and independent predictors of renal insufficiency. Table 2 shows the binary logistic regression results.

**Table 2:** Binary logistic regression results.

Parameters	Wald	B	SE	Odds ratio	95% CI		P value
					Lower	Upper	
History of hypertension	6.457	2.31	0.907	10.032	1.694	59.399	0.011
Vascular amyloid deposits	5.814	2.48	1.029	11.952	1.591	89.784	0.016
Percentage of globally sclerotic glomeruli	1.581	0.06	0.47	1.061	0.967	1.165	0.209

Regression equation: GFR <60 = -1.5 + 2.3 (presence of hypertension) + 2.5 (vascular amyloid deposits score ≥2). CI: confident interval; B: coefficient; SE: standard error; GFR: Glomerular filtration rate.

## Discussion

This paper aimed to investigate the impact of clinical and histological findings on renal survival of patients with biopsy-proven renal amyloidosis. For this, we studied a series of patients with renal amyloidosis in whom we observed a strong correlation between the degree of GFR and the presence of history of hypertension or the amount of amyloid deposits in renal small vessels. The statistically significant association between vascular amyloid deposits and worst GFR, a previously controversial finding in the literature, was the most intriguing point in our study. It would be worth knowing if these patients would have a worse prognosis, with a more rapid evolution to dialysis, renal transplantation, or even death. Clinical and morphological correlations related to renal amyloidosis have previously been reported, with some studies using quantitative or semiquantitative methods (17,20). These studies included the presence of vascular deposits but used different methods to estimate renal function, sometimes even without an estimated GFR (12,15,21) or presenting conflicting results in terms of statistical significance (9,13). Therefore, we do not have a substantial number of detailed and carefully-planned studies that address the distribution of amyloid deposits in the three renal compartments and their possible association with fundamental clinical variables such as GFR.

It was previously described that vascular deposits could correlate with high blood pressure (10), although we did not observe this correlation in our study. Others studies also reported that patients with amyloid confined to the tubulointerstitial tissue and vasculature presented lower levels of GFR (22). However, a study describing AA amyloid renal involvement in rheumatoid arthritis found no deterioration of serum creatinine and binding urea nitrogen (BUN) in a 5-year follow-up period when the pattern was exclusively vascular (14). We suspect that findings like vascular amyloid deposition could indicate disease activity in the context of renal amyloidosis. More traditional findings of chronicity acting as confounders like glomerulosclerosis degree, interstitial fibrosis percentage, and the arteriosclerosis index could be simply linked to aging or we must start facing renal amyloidosis also as a disease of small vessels, not only as a glomerular disease (21).

We emphasized here the importance of using e-GFR and the CKD stages when we aimed to understand the effects of deposits or other elements on renal function, a determining factor for understanding the degree of renal disease and patient severity. This allowed us to divide the patients into two groups according to CKD classification, above or below CKD stage III. The distribution among groups A and B did not obey a case-control study design, such that the choice of the GFR value that divided the two groups was empirical. Most cases with low e-GFR were closely correlated with high blood pressure, a well-known mechanism in chronic kidney disease progression (23). Longitudinal studies have observed that renal function was a weak predictor of prognosis and overall survival after the time of biopsy diagnosis (24,25), including the accelerated progression of renal dysfunction (26) and e-GFR >60 mL·min^-1^·(1.73 m^2^)^-1^ as a low-risk factor for a shorter survival (27). Regarding the diagnosis of AL or non-AL subtypes, we did not observe association with renal dysfunction. Similarly, no difference was observed between the survival curves of AL and AA patients and survival rate at 5 and 10 years was similarly small for both subtypes (17), although there are conflicting results in other studies with a great influence of the presence of end stage kidney disease (11,28–30).

Descriptive histopathological classifications and scores are important for kidney biopsy because clinicians usually expect not only the diagnosis itself, but also some parameters that could help in patient management. Unfortunately, in cases of renal amyloidosis, the extra glomerular compartments are not routinely detailed in terms of amyloid deposit extent or distribution, which may limit these possible clinical interpretations. This work highlighted the importance of describing amyloid deposit extent in the renal compartments and including these descriptions and scores in renal pathologists’ routine biopsy reports. Indeed, these ideas are not recent and some efforts have been already made. For example, glomerular renal amyloidosis classifications have been proposed, based on extension and distribution of amyloid deposits (20,31–33). However, these approaches have not yet been fully validated for clinical outcomes. Additionally, some studies have analyzed the amyloid distribution with regards to three patterns – predominant glomerular, vascular, or tubulointerstitial deposits (9), or mentioning only two patterns of deposit distribution – exclusively glomerular or vascular (12).

Sen and Sarsik created an interesting renal amyloidosis classification that echoes morphological aspects of the lupus nephritis classification, which also adopted a quantitative glomerular approach (20). They defined six glomerular involvement classes, from minimum mesangial deposits to diffuse mesangiocapillary pattern (they also included a membranous pattern as lupus nephritis class V). This classification is more descriptive, mainly based on glomeruli, and is sometimes static. However, it has not been validated for clinical outcomes and our focus was not on the morphological glomerular aspect, thus we did not use these parameters. We have adopted a simple and useful way to semi-quantify the kidney tissue, focusing on all three compartments (glomerular, tubulointerstitial, and vascular). Each was evaluated separately and following an increasing score according to the amyloid deposit amount, which allowed us to quantify each compartment. Moreover, we could then apply a statistical mathematical model to create inferences that could easily test a clinical hypothesis. Surprisingly, beyond the glomeruli, the vascular amyloid deposit is important when considering renal function as obtained by e-GFR.

In our study, information on history of hypertension was obtained from patient records during kidney biopsy. Obviously, it is unclear whether this history resulted from a disease prior to or secondary to amyloidosis. It is essential to emphasize here some conceptual points. A history of systemic arterial hypertension is often found in many advanced age individuals generally as an already-present comorbidity. Thus, arterial hypertension may have been present for some years even before the amyloidosis diagnosis. Nephrogenic hypertension related to loss of renal function may also be observed, which may therefore have an indirect association with renal amyloidosis (11). Hypotension in AL patients is much more frequent than in AA. It is usually due to orthostatic hypotension, which is mainly seen as a consequence of neural impairment or even of treatment (34–36). Still, only about 15% of patients with AL will develop neural changes (36,37). However, the kidney is one of the most affected organs in AL amyloidosis (as in AA) (26). From this perspective, few patients with AL-type renal amyloidosis will have hypotension because they will not necessarily have neural involvement along with renal involvement. Several studies of renal amyloidosis report a considerable percentage of hypertensive patients. This may be related to comorbidity in elderly individuals, nephrogenic origin in those with renal dysfunction, and even the presence of vascular deposits detailed in this study and only briefly cited in others (6,10,36,38). In our study, renal impairment stands out in both AL and AA, while neural impairment (a cause of hypotension in AL patients) occurs in a minority of cases, as observed in our series.

This is a relatively large series (53 cases) in which the inclusion criterion was the diagnosis of amyloidosis in renal biopsy and the main aim was to study the GFR decrease. This is a different situation from systemic amyloidosis seen in the internal medicine setting or even to cases with isolated nephrotic syndrome and preserved GFR. It is noteworthy that history of hypertension can be seen in our findings as a separate marker to the presence of vascular deposits concerning loss of renal function. Each marker has its own power to be an independent factor in predicting GFR loss, as observed in our statistical model. It’s important to emphasize that we have no evidence in this study concerning a direct correlation between vascular deposits and arterial hypertension. However, the question concerning the effect of vascular amyloid deposits interested us. We believe that renal biopsies reports should include this amyloid semiquantitative analysis of renal compartments. Thus, from then on, we would start a new way of approaching renal outcomes from the tissue and from the diagnosis of renal amyloidosis. This is our study’s biggest contribution: highlighting that longitudinal studies and renal outcomes of renal amyloidosis should be proposed, despite the difficulty of dealing with a relatively rare disease, which would require multicenter studies.

There are some limitations in our study, it was a retrospective case series, with a considerable number of patients. However, we faced an uncommon disease. Our study comprises most cases of amyloidosis diagnosed by renal biopsies reviewed by nephropathologists in our region in the last 18 years. Additionally, the database comprised most histopathological details, including semiquantitative analysis of the amyloid deposits per renal compartment and other findings such as the amyloid subtype, fibrosis, interstitial infiltrates, atherosclerosis, and glomerular sclerosis. We obtained good insights into key clinical nephrology findings, such as proteinuria, history of hypertension, hematuria and, most especially, an estimated GFR using the CKD-EPI formula. We only performed cross-sectional associations between clinical and pathological variables, so these findings need to be confirmed by larger longitudinal and prospective studies.

In conclusion, beyond promoting a diagnosis of amyloidosis and their subtypes, renal biopsy may also suggest prognostic factors closely correlated to renal function. Unlike other diseases such as lupus nephritis and IgA nephropathy, for which we have clear and clinically-related histological classification parameters, histopathological reports of renal amyloidosis could possibly indicate a specific prognosis, for example. This approach could provide new insights and highlight more clinically-useful information, such as the fact that vascular amyloid deposits are independent predictors of renal dysfunction in cases of renal amyloidosis.

## References

[1] Wechalekar AD, Gillmore JD, Hawkins PN (2016). Systemic amyloidosis. Lancet.

[2] Briki F, Verine J, Doucet J, Benas P, Fayard B, Delpech M (2011). Synchrotron x-ray microdiffraction reveals intrinsic structural features of amyloid deposits in situ. Biophys J.

[3] Bergesio F, Ciciani AM, Santostefano M, Brugnano R, Manganaro M, Palladini G (2007). Renal involvement in systemic amyloidosis--an Italian retrospective study on epidemiological and clinical data at diagnosis. Nephrol Dial Transplant.

[4] Tsai SF, Wen MC, Cheng CH, Wu MJ, Chen CH, Yu TM (2011). Clinical features of renal amyloidosis: an analysis of 40 patients in a 28-year follow-up. Intern Med.

[B05] von Hutten H, Mihatsch M, Lobeck H, Rudolph B, Eriksson M, Rocken C (2009). Prevalence and origin of amyloid in kidney biopsies. Am J Surg Pathol.

[6] Abdallah E, Waked E (2013). Incidence and clinical outcome of renal amyloidosis: a retrospective study. Saudi J Kidney Dis Transpl.

[7] Polito MG, de Moura LA, Kirsztajn GM (2010). An overview on frequency of renal biopsy diagnosis in Brazil: clinical and pathological patterns based on 9,617 native kidney biopsies. Nephrol Dial Transplant.

[8] da Fonseca EO, Filho PJ, da Silva LE, Caldas ML (2015). Epidemiological, clinical and laboratorial profile of renal amyloidosis: a 12-year retrospective study of 37 cases. J Nephropathol.

[9] Hopfer H, Wiech T, Mihatsch MJ (2011). Renal amyloidosis revisited: amyloid distribution, dynamics and biochemical type. Nephrol Dial Transplant.

[10] Dember LM (2006). Amyloidosis-associated kidney disease. J Am Soc Nephrol.

[11] Pettersson T, Konttinen YT (2010). Amyloidosis-recent developments. Semin Arthritis Rheum.

[12] Shiiki H, Shimokama T, Yoshikawa Y, Toyoshima H, Kitamoto T, Watanabe T (1988). Renal amyloidosis. Correlations between morphology, chemical types of amyloid protein and clinical features. Virchows Arch A Pathol Anat Histopathol.

[13] Verine J, Mourad N, Desseaux K, Vanhille P, Noel LH, Beaufils H (2007). Clinical and histological characteristics of renal AA amyloidosis: a retrospective study of 68 cases with a special interest to amyloid-associated inflammatory response. Hum Pathol.

[14] Uda H, Yokota A, Kobayashi K, Miyake T, Fushimi H, Maeda A (2006). Two distinct clinical courses of renal involvement in rheumatoid patients with AA amyloidosis. J Rheumatol.

[15] Watanabe T, Saniter T (1975). Morphological and clinical features of renal amyloidosis. Virchows Arch A Pathol Anat Histol.

[16] Looi LM, Cheah PL (1997). Histomorphological patterns of renal amyloidosis: a correlation between histology and chemical type of amyloidosis. Hum Pathol.

[17] Sasatomi Y, Sato H, Chiba Y, Abe Y, Takeda S, Ogahara S (2007). Prognostic factors for renal amyloidosis: a clinicopathological study using cluster analysis. Intern Med.

[18] KDIGO (2013). Summary of Recommendation Statements. Kidney Int Suppl (2011).

[19] Zanocco JA, Nishida SK, Passos MT, Pereira AR, Silva MS, Pereira AB (2012). Race adjustment for estimating glomerular filtration rate is not always necessary. Nephron Extra.

[20] Sen S, Sarsik B (2010). A proposed histopathologic classification, scoring, and grading system for renal amyloidosis: standardization of renal amyloid biopsy report. Arch Pathol Lab Med.

[21] Castano E, Palmer MB, Vigneault C, Luciano R, Wong S, Moeckel G (2015). Comparison of amyloid deposition in human kidney biopsies as predictor of poor patient outcome. BMC Nephrol.

[22] Kidd J, Carl DE (2016). Renal amyloidosis. Curr Probl Cancer.

[23] Griffin KA (2017). Hypertensive kidney injury and the progression of chronic kidney disease. Hypertension.

[24] Kyle RA, Gertz MA (1995). Primary systemic amyloidosis: clinical and laboratory features in 474 cases.. Semin Hematol.

[25] Bohle A, Wehrmann M, Eissele R, von Gise H, Mackensen-Haen S, Muller C (1993). The long-term prognosis of AA and AL renal amyloidosis and the pathogenesis of chronic renal failure in renal amyloidosis. Pathol Res Pract.

[26] Osawa Y, Kawamura K, Kondo D, Imai N, Ueno M, Nishi S (2004). Renal function at the time of renal biopsy as a predictor of prognosis in patients with primary AL-type amyloidosis. Clin Exp Nephrol.

[27] Ozawa M, Komatsuda A, Ohtani H, Nara M, Sato R, Togashi M (2017). Long-term prognosis of AL and AA renal amyloidosis: a Japanese single-center experience. Clin Exp Nephrol.

[28] Tang W, McDonald SP, Hawley CM, Badve SV, Boudville N, Brown FG (2013). End-stage renal failure due to amyloidosis: outcomes in 490 ANZDATA registry cases. Nephrol Dial Transplant.

[B29] Bergesio F, Ciciani AM, Manganaro M, Palladini G, Santostefano M, Brugnano R (2008). Renal involvement in systemic amyloidosis: an Italian collaborative study on survival and renal outcome. Nephrol Dial Transplant.

[30] Gertz MA, Lacy MQ, Dispenzieri A (2002). Immunoglobulin light chain amyloidosis and the kidney. Kidney Int.

[31] Kuroda T, Tanabe N, Kobayashi D, Wada Y, Murakami S, Nakano M (2012). Significant association between renal function and amyloid-positive area in renal biopsy specimens in AL amyloidosis. BMC Nephrol.

[B32] Kuroda T, Tanabe N, Kobayashi D, Wada Y, Murakami S, Nakano M (2012). Significant association between renal function and area of amyloid deposition in kidney biopsy specimens in reactive amyloidosis associated with rheumatoid arthritis. Rheumatol Int.

[33] Kuroda T, Ito Y, Imai N, Nozawa Y, Sato H, Nakatsue T (2017). Significant association between renal function and area of amyloid deposition evident in kidney biopsy specimens in both AA and AL amyloidosis. Amyloid.

[34] Bollée G, Guery B, Joly D, Snanoudj R, Terrier B, Allouache M (2008). Presentation and outcome of patients with systemic amyloidosis undergoing dialysis. Clin J Am Soc Nephrol.

[B35] Merlini G, Comenzo RL, Seldin DC, Wechalekar A, Gertz MA (2014). Immunoglobulin light chain amyloidosis. Expert Rev Hematol.

[36] Nishi S, Muso E, Shimizu A, Sugiyama H, Yokoyama H, Ando Y (2017). A clinical evoluation of renal amyloidosis in the Japan renal biopsy registry: a cross-sectional study. Clin Exp Nephrol.

[37] Abdallah E, Waked E (2013). Incidence and clinical outcome of renal amyloidosis: a retrospective study. Saudi J Kidney Dis Transpl.

[38] Nasr SH, Valeri AM, Sethi S, Fidler ME, Cornell LD, Gertz MA (2012). Clinicopathologic correlations in multiple myeloma: a case series of 190 patients with kidney biopsies. Am J Kidney Dis.

